# Relationship between agricultural biodiversity and dietary diversity of children aged 6-36 months in rural areas of Northern Ghana

**DOI:** 10.1080/16546628.2017.1391668

**Published:** 2017-10-27

**Authors:** Mahama Saaka, Shaibu Mohammed Osman, Irmgard Hoeschle-Zeledon

**Affiliations:** ^a^ School of Allied Health Sciences, University for Development Studies, Tamale, Ghana; ^b^ Department of Research for Development, International Institute of Tropical Agriculture (IITA), Ibadan, Nigeria

**Keywords:** Agrobiodiversity, preschool children, socioeconomic status, causal mediation, interaction, Northern Ghana

## Abstract

In this study, we investigated the relationship between agricultural biodiversity and dietary diversity of children and whether factors such as economic access may affect this relationship.

This paper is based on data collected in a baseline cross-sectional survey in November 2013.The study population comprising 1200 mother-child pairs was selected using a two-stage cluster sampling. Dietary diversity was defined as the number of food groups consumed 24 h prior to the assessment. The number of crop and livestock species produced on a farm was used as the measure of production diversity. Hierarchical regression analysis was used to identify predictors and test for interactions.

Whereas the average production diversity score was 4.7 ± 1.6, only 42.4% of households consumed at least four food groups out of seven over the preceding 24-h recall period. Agricultural biodiversity (i.e. variety of animals kept and food groups produced) associated positively with dietary diversity of children aged 6–36 months but the relationship was moderated by household socioeconomic status. The interaction term was also statistically significant [β = −0.08 (95% CI: −0.05, −0.01, p = 0.001)].

Spearman correlation (rho) analysis showed that agricultural biodiversity was positively associated with individual dietary diversity of the child more among children of low socioeconomic status in rural households compared to children of high socioeconomic status (r = 0.93, p < 0.001 versus r = 0.08, p = 0.007). Socioeconomic status of the household also partially mediated the link between agricultural biodiversity and dietary diversity of a child’s diet.

The effect of increased agricultural biodiversity on dietary diversity was significantly higher in households of lower socioeconomic status. Therefore, improvement of agricultural biodiversity could be one of the best approaches for ensuring diverse diets especially for households of lower socioeconomic status in rural areas of Northern Ghana.

## Introduction

It has been estimated that 795 million people suffer from undernutrition worldwide [1] and about 780 million of these live in developing countries during the period 2014–16 [2].When properly linked, agrobiodiversity, agriculture and nutrition constitute a common galvanizing approach to attaining food and nutrition security, an important goal of the Sustainable Development Goals (SDGs). A large proportion of an estimated 2 billion people suffer from low intakes of vitamins and minerals such as iron and zinc [3]. These nutritional deficiencies are a result of low food quantities consumed and/or poor dietary quality. Diversified agricultural production is most likely to provide a wide range of different types of foods to poor population segments [4].

Agrobiodiversity is defined as the variety and variability of animals, plants and micro-organisms used directly or indirectly for food and agriculture [5]. Biodiversity ensures sustainability and resilience within the food system [6–8]. Furthermore, available scientific evidence demonstrates that biodiversity is important to human health and nutrition including the provision of macronutrients, micronutrients, and bioactive non-nutrients for healthy diets [9–11].

A number of organizations including the World Health Organization (WHO), the Food and Agriculture Organization (FAO) of the United Nations, the Consultative Group on International Agricultural Research (CGIAR) and HarvestPlus have advocated for dietary diversity strategies to tackle the burden of micronutrient malnutrition [12–14]. This is based on empirical evidence which suggests that dietary diversity is a proxy of diet quality and the consumption of a variety of foods across and within food groups and across different varieties of specific foods is a precondition for adequate intake of essential nutrients [15–19].

If households consume what they produce, it is logical to expect that households that have diversified crops and animals should have diversified diets and so diverse farm production has been encouraged as a means to increase dietary diversity [9,20]. However, empirical evidence on the link between production and consumption diversity appears inconclusive and may depend on other factors[21,22]. In particular, more research is needed to better understand in which socioecological settings and contexts do biodiversity and nutritional and dietary outcomes relate and which factors mediate the relationship. In this study, we investigated the relationship between agricultural biodiversity and dietary diversity of children and whether factors such as economic access may mediate this relationship.

## Materials and methods

### Study area

The study was undertaken in resource-poor households in rural areas of Northern Ghana where the primary occupation is farming. The study area is characterized by high poverty and recurrent droughts and floods which predispose communities to increased vulnerability to food insecurity and malnutrition. The Ghana Living Standards Survey Round 6 Report showed that the regions where the study was conducted have higher proportions of households in the lowest quintile than in the highest quintile [23].

The majority of the people have agriculture as their main occupation while some are involved in trading. The main staple foods including maize, sorghum, millet and yam are usually harvested from October through December. Although the food security situation is usually good during harvest time, child care tends to suffer because of lack of time on the part of rural mothers. A high proportion of rural mothers work daily away from home, and therefore frequently face challenges to the care of children.

The rainfall pattern is unimodal and the period is usually short and lasts from May to August, followed by a long dry season (September – April) with dry harmattan winds.

### Survey design, population and sampling

This paper is based on analysis of data which were collected in a baseline survey prior to an intervention study. The intervention package focused on nutrition behavior change communication (BCC) for improved child and maternal nutrition. In the intervention communities where the International Institute of Tropical Agriculture (IITA) developed and promoted better agronomic practices, they received a nutrition education package in addition to routine health services. The educational sessions were mainly messages that promote better health and nutrition, focusing on 1) appropriate complementary feeding such as use of thicker instead of thinner porridges; 2) use of animal-source foods; 3) dietary diversity; and 4) personal hygiene.

In the comparison communities, eligible households were those that had no previous exposure to IITA program activities but they also received general health and nutrition messages at monthly growth monitoring sessions.

The baseline survey report has been reported elsewhere [24] but, briefly, a community-based cross-sectional cluster survey was carried out in November 2013. The study population comprised mothers/primary caregivers and their children. A stratified, two-stage sample design in which the primary sampling units (communities) were selected with probability proportional to size within each of the five districts was used. Households were selected using random systematic sampling within each cluster. In each selected cluster, a complete list of all households was compiled, and systematic random sampling was used to select eligible households.

The primary outcome variable used to estimate the sample size was the population proportion of chronic malnutrition (25.0 %) in the study area (Nutrition Surveillance Report, 2013, Unpublished). This outcome indicator was used to calculate a sample size of 1200 (600 per intervention and comparison areas). A sample size of 288 was required to ensure that the estimated prevalence of the main outcome variable was within plus or minus 5% of the true prevalence at 95% confidence level. Assuming a correction factor of 2 (the ‘design effect’) for cluster sampling, the sample size was increased to 576. A non-response rate of 5% and other unexpected events (e.g. damaged/incomplete questionnaire) was factored in the sample size determination and so the sample size is adjusted to 600 for 25 intervention communities. The same number of children was selected from comparison communities using probability proportionate to size (PPS).

The Emergency Nutrition Assessment (ENA) software was used to randomly select the required number of clusters.

### Measurement of variables

The main outcome variable for this study was dietary diversity score of households and farm production diversity as an explanatory variable. The independent covariates were maternal, child and household characteristics. Child’s age was categorized into 6–8 months, 9–11 months, 12–23 months and 24–36 months. A brief description of main independent and dependent variables is as follows:

### Measurement of agricultural biodiversity and dietary diversity

As in previous studies, agricultural biodiversity was measured by the number of food groups grown and/or types of animals raised for food [25–27]. Households recalled all food groups and livestock grown/reared during the previous agricultural season were collected from both mother and father in each household through interviews. Agricultural biodiversity score at the household level was therefore calculated by summing the number of food groups and/or types of animals raised for food and sale. If a household produces several varieties of food crops that belong to the same food groups, the production diversity score will be smaller than the simple species count.

Agricultural production diversity was also categorized (livestock only, crops only, crops and livestock, and nothing) and tested for association against minimum dietary diversity.

Dietary diversity of the child was measured as per WHO guidelines [28,29]. The seven foods groups used for calculation of WHO minimum dietary diversity indicator are:

1) grains, roots and tubers; 2) legumes and nuts; 3) dairy products; 4) flesh foods; 5) eggs; 6) vitamin A rich fruits and vegetables; and 7) other fruits and vegetables.

The dietary diversity score (DDS) was calculated by summing the number of food groups consumed by the child as reported over the 24-h recall period. From the dietary diversity score, the minimum dietary diversity indicator was constructed. Minimum dietary diversity is the proportion of children who ate at least four or more varieties of foods from the seven food groups in a 24-h time period [28,29].

### Determination of household economic status

A household wealth index based on household assets and housing quality was used as a proxy indicator for socioeconomic status (SES) of households. Principal Component Analysis (PCA) was used to determine household wealth index from information collected on housing quality (floor, walls, and roof material), source of drinking water, type of toilet facility, the presence of electricity, type of cooking fuel, and ownership of modern household durable goods (e.g. bicycle, television, radio, motorcycle, sewing machine, telephone, cars, refrigerator, mattress, bed, computer and mobile phone) [30–33].

### Data processing and analysis

The analysis of data took into account the complex design of multi-stage cluster surveys. All quantitative data were coded for statistical analysis using SPSS Complex Samples module for Windows 18.0 (SPSS Inc., Chicago). This was done in order to make statistically valid population inferences and computed standard errors from sample data. Design weights were added to each district’s sample data (i.e. total population divided by number of respondents) to perform weighted analysis.

Bivariate associations were made between agricultural biodiversity and individual dietary diversity of children using Spearman rank correlation coefficients.

We conducted three-step moderated hierarchical multiple regression analyses to determine independent predictors and moderators of dietary diversity of the child. Multicollinearity was investigated by using the variance inflation factor (VIF). A VIF (the reciprocal of the tolerance statistics) of greater than 5 is generally considered evidence of multicollinearity.

Potential effect modification (statistical interaction) was investigated to ascertain whether the relationship between agricultural biodiversity and individual dietary diversity of children was moderated by socioeconomic status of household. Effect modification was identified and adjusted for through using three-step moderated hierarchical multiple regression analyses.

The main covariate predictor variables (household wealth index, age group of child and household size) were entered in the first step. In the second step we added the main explanatory variable of interest (i.e. agricultural production diversity) and the interaction term (moderation) was added in the third step. The interaction term comprised the product of the centered agrobiodiversity score and centered household wealth index.

Also, mediation analysis which provides a better understanding of the causal chain by which an independent variable (X) influences a dependent variable (Y) through a mediator (M) [34] was used to assess whether socioeconomic status of the household mediates the link between agricultural biodiversity and dietary diversity of a child’s diet.

### Ethical considerations

The study protocol was approved by the Scientific Review Committee of the School of Allied Health Sciences, University for Development Studies, Ghana. Ethics clearance was obtained from the Institutional Review Board (IRB) of the Tamale Teaching Hospital, Ghana (Ref no. TTH/10/11/15/01). Participation in the study was voluntary and no incentives were provided. Verbal informed consent was sought from all the study participants before the commencement of any interview. The study was not harmful to any study participant. Study participants were free to withdraw from the study at any time without any penalty.

## Results

### Sample characteristics

Table 1 presents the summary statistics on key characteristics of mother – child pairs in our sample.

**Table 1.** Sample characteristics (*n* = 1200).

	Frequency (n)	Percentage (%)
**Mothers’ Age Groups (years)**		
Under 18	13	1.1
18–35	982	81.8
35^+^	205	17.1
**Educational level**		
None	845	70.4
Primary	189	15.8
Junior High School (JHS)	133	11.1
Senior High School (SHS)	28	2.3
Tertiary (College/university)	5	0.4
**Region of residence**		
Northern	480	40.0
Upper West	478	39.8
Upper East	242	20.2
**Children under five years in Household**		
1–2	896	74.7
3–4	232	19.3
More than 4	72	6.0
**Source of food**		
Own production	1,110	92.5
Purchases	90	7.5
**Who takes decisions about purchasing food?**		
Mother/caregiver	256	21.3
Husband/partner	595	49.6
Mother/caregiver and partner	253	21.1
Other older person in household/family	96	8.0

A total of 1200 mothers/caretakers were interviewed at the household level. The mean age of the respondents was 29.2 ± 6.7 years and a majority of them (81.8%) were in the age group of 18–35 years.

The mean number of children under five years of age living a household was 2.0 ± 1.3 with a range of 1–10. The majority of respondents, 70.4% (845), had no formal education at all.

More than 90% of foods consumed by households were their own production and 49.6% of husbands/partners made the decision on how much money was spent on food.

### Agricultural biodiversity and dietary diversity

Whereas the average production diversity score was 4.7 ± 1.6, only 42.4% of households consumed at least four food groups out of seven over the two preceding 24-h recall periods. In terms of both crop production diversity (CPD) and livestock production diversity (LPD), most households were producing 1–2 crop/livestock groups (Table 2).

**Table 2.** Proportion of households growing food crops and rearing animals (*n* = 1200).

No. of crop/livestock varieties produced		Proportion of households growing food crops and rearing animals	
	Mean ± SD	Frequency (n)	Percentage (%)
Food crop production diversity (no. of food crop groups produced	2.4 ± 0.85		
Livestock production diversity	2.2 ± 1.13		
Production diversity (no. of crop/livestock groups produced)	4.7 ± 1.64		
Dietary diversity score (no. of food groups consumed)	3.1 ± 1.62		
**Crop Production Diversity (CPD)**			
0 (Nothing)		18	1.5
1–2		685	57.1
3–4		491	40.9
> 4		6	0.5
**Livestock Production Diversity (LPD)**			
0 (Nothing)		71	5.9
1–2		676	56.3
3–4		448	37.3
> 4		5	0.4
**Production Diversity (PD)**			
0 (Nothing)		11	0.9
1-2		86	7.2
3–4		478	39.8
> 4		625	52.1
**Minimum dietary diversity**			
Less than 4 groups		691	57.6
At least 4 groups		509	42.4

### Relationship between agricultural biodiversity and dietary diversity

Spearman rank correlation (Spearman’s rho) analysis in Table 3 shows that livestock production diversity, crop production diversity and overall production diversity significantly correlated with dietary diversity.

**Table 3.** Association between production diversity and dietary diversity.

		Crop production diversity	Livestock production diversity	Production diversity	Dietary diversity score
Crop production diversity	Spearman *R*	1	0.37^a^	0.74^a^	0.10^a^
	Sig. (2-tailed)		<0.001	<0.001	0.01
	*n*	1200	1200	1200	1200
Livestock production diversity	Spearman *R*	0.37^a^	1	0.89^a^	0.10^a^
	Sig. (2-tailed)	<0.001		<0.001	0.002
	*n*	1200	1200	1200	1200
Production diversity	Spearman *R*	0.74^a^	0.89^a^	1	0.12^a^
	Sig. (2-tailed)	<0.001	<0.001		<0.001
	*n*	1200	1200	1200	1200

^a^ Correlation is significant at the 0.01 level (2-tailed).

### Moderation effects of socio-economic status of household on production diversity in predicting dietary diversity score for children aged 6–36 months

In step 2 of the moderated hierarchical multiple regression analyses, when the explanatory variable (i.e. production diversity) was added to the regression model, the percentage of variability accounted for went up from 20.1% to 20.9% (R^2^ Change = 0.008, p < 0.001) (Table 4).

**Table 4.** Regression model summary.

Model	R	R Square	Adjusted R Square	Std. Error of the Estimate	Change Statistics				
					R Square Change	F Change	df1	df2	Sig. F Change
1	0.45^a^	0.203	0.201	1.44756	0.203	99.581	3	1176	<0.001
2	0.46^b^	0.212	0.209	1.43977	0.008	13.761	1	1175	<0.001
3	0.47^c^	0.219	0.215	1.43397	0.007	10.515	1	1174	0.001

^a^Predictors: (Constant), Classification of child’s age, Classification of principal components, Household size.

^b^Predictors: (Constant), Classification of child’s age, Classification of principal components, Household size, Production diversity.

^c^Predictors: (Constant), Classification of child’s age, Classification of principal components, Household size, Production diversity, Interaction term (Production diversity x wealth).

Biodiversity associated with dietary diversity of children but the relationship was moderated by household socioeconomic status. The interaction term added significantly beyond the main effects (R^2^ Change = 0.007, p = 0.001), indicating that there was a statistically significant interaction between household socioeconomic status and production diversity in predicting dietary diversity scores.

### Relationship between agricultural biodiversity and dietary diversity

There was a positive relationship between agricultural biodiversity (variety of animals kept and plants grown for food) and the diversity of a child’s diet. Table 5 shows ‘coefficients’ with the predictors which were statistically significant. Older children (24–36 months), high agricultural biodiversity, large household size and households of higher socioeconomic status household wealth index were consistent predictors. The set of variables accounted for 21.5% of the variance in dietary diversity, the dependent variable  based on WHO food groupings. (Adjusted R square = 20.215).

**Table 5.** Predictors of dietary diversity score for children aged 6–36 months.

Model	Covariates	Unstandardized Coefficients		Standardized Coefficients	T	Sig.	95.0% Confidence Interval for β		Collinearity Statistics	
		B	Std. Error	Beta (β)			Lower Bound	Upper Bound	Tolerance	VIF
1	(Constant)	0.37	0.20		1.83	0.07	−0.03	0.77		
	Larger household size	0.16	0.07	0.06	2.25	0.02	0.02	0.31	1.00	1.00
	High wealth index	0.36	0.12	0.08	2.96	0.003	0.12	.590	.999	1.001
	Older child’s age group	0.75	0.04	0.45	17.05	<0.001	0.66	0.84	1.00	1.003
2	(Constant)	−0.03	0.23		−0.13	0.89	−0.48	0.42		
	Larger household size	0.16	0.07	0.06	2.19	0.03	0.02	0.30	1.00	1.002
	High wealth index	0.30	0.12	0.07	2.52	0.01	0.07	0.54	0.99	1.02
	Older child’s age group	0.75	0.04	0.45	17.18	<0.001	0.67	0.84	1.00	1.003
	Production diversity score	0.10	0.03	0.10	3.71	<0.001	0.05	0.15	0.99	1.02
3	(Constant)	0.02	0.23		0.10	0.92	−0.43	0.47		
	Larger household size	0.16	0.07	.056	2.16	0.03	0.01	0.30	1.00	1.002
	High wealth index	0.26	0.12	0.06	2.18	0.03	0.03	0.50	0.98	1.026
	Older child’s age group	0.75	0.04	0.45	17.27	<0.001	0.67	0.839	1.00	1.003
	Production diversity score	0.09	0.03	0.09	3.62	<0.001	0.04	0.144	0.99	1.02
	Interaction term (Production diversity x wealth)	−0.03	0.01	−0.08	−3.24	0.001	−0.05	−0.01	0.99	1.01

The strongest predictor was child’s age with a standardized beta (β) weight of 0.45, p < 0.001. The second highest contributor was production diversity with beta (β) weight of 0.09, p < 0.001.

Producing one additional crop or livestock species leads to a 0.09 standard units (9%) increase in the number of food groups consumed.

The interaction term was also statistically significant [β = −0.08 (95% CI −0.01‒0.05, p = 0.001] and this confirms that the relationship between production diversity and dietary diversity scores differs according to the socioeconomic status of the child’s household.

The slopes of the regression lines between production diversity and dietary diversity scores will therefore be different for the different categories of socioeconomic status by −0.08. This negative slope gives an indication that the effect of production diversity on dietary diversity is lower in households of higher socioeconomic status. The effect was significantly higher among households of low wealth index.

Similarly, Spearman correlation (rho) analysis showed that agricultural biodiversity was positively associated with individual dietary diversity of the child more among children of low socioeconomic status in rural households compared to children of high socioeconomic status (r = 0.93, p < 0.001) vs. (r = 0.08, p = 0.007).

Table 6 presents the results of the mediation analysis. From the analyses, socioeconomic status of the household partially mediated the link between agricultural biodiversity and dietary diversity of a child’s diet.

**Table 6.** Causal mediation analysis.

Path	Beta (β)	t values	P-value	95.0% Confidence Interval for β		Interpretation
				Lower Bound	Upper Bound	
1.Agricultural biodiversity (X) predicting dietary diversity (Y)	0.10	3.39	0.001	0.04	0.15	There is relationship between X and Y
2.Agricultural biodiversity (X) predicting wealth index (M)	0.120	4.181	<0.001	0.10	0.27	X and M have relationship and so mediation makes sense
3. Wealth index (M) predicting dietary diversity (Y)	0.041	1.42	0.157	−0.01	0.07	Effect of M is insignificant
4.Agricultural biodiversity (X) and wealth index (M) predicting dietary diversity (Y)	0.09	3.18	0.001	0.04	0.15	Effect of X is weakened after controlling for M.
	0.03	1.03	0.30	−0.02	0.06	

## Discussion

Although some evidence suggests that agricultural biodiversity is strongly associated with dietary diversity, the mediation effects of socioeconomic status on dietary quality remain unclear. In this paper we investigated whether and how biodiversity contributes to diversified diets of children and how economic factors interact with biodiversity to influence consumption of diversified diets.

### Determinants of dietary diversity of children

In the present study, the principal determinants of increased dietary diversity for children were age of child, high wealth index, larger household size and increased agricultural biodiversity. The dietary quality of an individual may be affected by a multiplicity of factors, such as food availability, preferences, household environment, and socioeconomic status [35,36].

Both Pearson correlation and hierarchical multiple regression analyses showed that livestock production diversity, crop production diversity and overall production diversity significantly and positively associated with dietary diversity of children aged 6–36 months. Biodiversity associated with dietary diversity of children, but the relationship was moderated by the socioeconomic status of the household. This finding is consistent with other studies that have shown a strong positive relationship between biodiversity in agricultural production and improved diversified diets [37–40]. The links between biodiversity and dietary diversity at the household or individual levels, however, remain inconclusive. This is because some studies have reported no association [41–44]. Results from a study in Tanzania show that at the bivariate data analysis level, dietary diversity was significantly and positively influenced by crop count but did not show a significant association with the Simpson’s Index which measures the number of crops as well as the distribution of area cultivated under various crops. However, farm crop diversity did not have a positive and significant effect on dietary diversity after controlling for other covariates [45].

In our study, production diversity that includes both crops and livestock was the main explanatory variable, whereas the study in Tanzania focused on only on-farm crop diversity (staples and vegetables). Also, the relationship between production diversity and dietary diversity may depend on whether food crop species or food groups were used in measuring production diversity. Usually, dietary diversity (DD) is measured as the number of food groups consumed and so it would be better to measure production diversity using food groups, as was done in this present study. Studies that have quantified production diversity using number of crop and livestock species produced on a farm may be over-estimating the production diversity. From the nutritional standpoint, increased production diversity means foods from various food groups. A farmer who grows maize, millet, sorghum in terms of diversity will be given a score of 1 but a farmer who grows maize and groundnuts will be given a score of 2. The variation in the measurement of production diversity may account for inconsistent findings regarding production and dietary diversity relationship.

Evidence of the relationship between biodiversity and nutrition, however, appears to be growing. Evidence from recent relevant studies [39,46–48] derived from different African and Asian countries showed that farm production diversity is positively associated with dietary diversity.

Therefore, an appropriate mix of behaviour change communication and production of local food varieties and poultry resources could be a feasible option to enhance recommended infant feeding practices and reduce under-nutrition since households that own small livestock, keep chickens, ducks, or other birds; for the meat/sale are more likely to provide children with diversified foods [49,50].

Increasing household size was significantly associated with increases in dietary diversity scores. Children in households comprising more adults were more likely to meet minimum dietary diversity standards. This may be explained by the fact that adults could be contributing to food access in the households through their own production or through purchases. These findings lend support to findings of previous studies [48,51].

### The role of socioeconomic status on the link between agricultural biodiversity and dietary diversity of children aged 6–36 months

Although some evidence suggests that agricultural biodiversity is strongly associated with dietary diversity [4,52–55], the socioecological settings and contexts in which this relationship exists are not fully established. In this study, socioeconomic status interacted with agricultural biodiversity on dietary diversity of a child, suggesting that the relationship between production diversity and dietary diversity scores differs according to the socioeconomic status of the child’s household. The interaction term between biodiversity and socioeconomic status as measured by household wealth index was significant (Table 5). The negative and significant interaction coefficient in the model suggests that the role of production diversity was more important in households of low socioeconomic status. For households of a lower socioeconomic position, production diversity increased the dietary diversity (the number of different food items consumed in the past 24 h) of the child.

This finding implies that when agricultural production is diversified, so that a wide range of different types of foods are available and accessible, then the poor population segments stand to benefit more than the well-to-do families. This is expected because in poor rural settings, the ability to access variety of foods through purchases may be limited. For such families the only sure way of obtaining diversified diets is through their own production.

This positive relationship between agrobiodiversity at the household level and dietary diversity is plausible, because what smallholder farmers produce is consumed at home [56]. However, this relationship may not be all that simple as smallholder subsistence farmers do also sell the food they produce in order to meet other pressing needs.

Socioeconomic status of the household also partly mediated the link between agricultural biodiversity and dietary diversity of children aged 6–36 months. The presence of the partial mediation effect of socioeconomic status of the household suggests the direct effect of agricultural biodiversity on dietary diversity had at least weakened. The direct effect of agricultural biodiversity on dietary diversity is most likely to be influenced by other factors such as socioeconomic status. Economic access to animal-source foods or other nutrient-rich foods such as fruits and vegetables may therefore contribute to improved dietary diversity [57–59].

## Conclusions

In this study, agricultural biodiversity showed a significant positive association with dietary diversity but its effect was moderated by the economic status of the household. The effect of production diversity on dietary diversity was significantly higher in households of lower socioeconomic status. Socioeconomic status of the household also partially mediated the link between agricultural biodiversity and dietary diversity of child’s diet.

Therefore, improvement of agricultural biodiversity could be one of the best approaches for ensuring diverse diets especially for households of lower socioeconomic status in rural areas of Northern Ghana.

Agricultural research and policy efforts that focus on an aggressive promotion of home gardens, poultry keeping and small livestock rearing especially among poor households is needed to improve dietary diversity.
